# Epidemiology and Genetic Characterization of Measles Strains in Senegal, 2004-2013

**DOI:** 10.1371/journal.pone.0121704

**Published:** 2015-05-22

**Authors:** Ndongo Dia, Ameth Fall, Rouguiyatou Ka, Amary Fall, David E. Kiori, Deborah G. Goudiaby, Aichatou D. Fall, El Hadj Abdourahmane Faye, Annick Dosseh, Kader Ndiaye, Ousmane M. Diop, Mbayame Nd. Niang

**Affiliations:** 1 Institut Pasteur de Dakar, Unité de Virologie Médicale, Dakar, Sénégal; 2 Laboratoire de Bactériologie, Centre Hospitalier National Universitaire de Fann, Dakar, Sénégal; Centers for Disease Control and Prevention, UNITED STATES

## Abstract

**Background:**

In Senegal, with the variable routine vaccination coverage, the risk for illness and death from measles still exists as evidenced by the measles epidemic episode in 2009. Since 2002 a laboratory-based surveillance system of measles was established by the Ministry of Health and the Institut Pasteur de Dakar. The present study analysed the data collected over the 10 years inclusive between 2004-2013 in order to define a measles epidemiological profile in Senegal, and we carried out a phylogenetic analysis of measles virus circulating in Senegal over the period 2009-2012.

**Methodology and Results:**

A total number of 4580 samples were collected from suspected cases, with the most cases between 2008 and 2010 (2219/4580; 48.4%). The majority of suspected cases are found in children from 4-6 years old (29%). 981 (21.4%) were measles laboratory-confirmed by IgM ELISA. The measles confirmation rate per year is very high during 2009-2010 periods (48.5% for each year). Regarding age groups, the highest measles IgM-positivity rate occurred among persons aged over 15 years with 39.4% (115/292) followed by 2-3 years old age group with 30.4% (323/1062) and 30% (148/494) in children under one year old group. The majority of suspected cases were collected between February and June and paradoxically confirmed cases rates increased from July (77/270; 28.6%) and reached a peak in November with 60% (93/155). Phylogenetic analysis showed that all the 29 sequences from strains that circulated in Senegal between 2009 and 2012 belong to the B3 genotype and they are clustered in B3.1 (2011-2012) and B3.3 (2009-2011) sub-genotypes according to a temporal parameter.

**Conclusion:**

Improvements in the measles surveillance in Senegal are required and the introduction of oral fluid and FTA cards as an alternative to transportation of sera should be investigated to improve surveillance. The introduction of a national vaccine database including number of doses of measles-containing vaccine will greatly improve efforts to interrupt and ultimately eliminate measles virus transmission in Senegal.

## Background

Measles virus is a ribonucleic acid (RNA) virus in the genus *Morbillivirus* within the family *Paramyxoviridae*. Although other members of the genus infect various animal species, measles only infect humans and non-human primates [1]. Measles virus (MV) is serologically monotypic, but analysis of the variability in the nucleotide sequences of wild-type viruses has identified a great diversity with eight clades (A—H), which have been divided into 22 genotypes and one proposed genotype. Clades B, C, D, G and H each contain multiple genotypes (B1–3, C1–2, D1–10, G1–3 and H1–2) while clades A, E and F each contains a single genotype (A, E and F) [2,3].

Measles disease is a highly contagious viral infection, most often with benign course. Its main complications are diarrhoea, acute otitis, viral or bacterial pneumonia; post-acute measles encephalitis usually occurs in 1–2 weeks after the rash. Sub-acute sclerosing panencephalitis (SSPE) is an extremely rare but invariably fatal, chronic, progressive encephalitis, that results from a measles virus infection acquired earlier in life. It is a major cause of infant morbidity and mortality worldwide, mainly in developing countries. For example in 2005, 345,000 deaths were reported, 87% of which were in the African and Asian Regions of World Health Organization (WHO) [4].

However during the last years, strategies based mainly on vaccination program have been implemented in order to reduce measles mortality. Considering the WHO regions, the highest percentage of reduction was in the Eastern Mediterranean (90%) and the African (89%) regions, accounting for 16 and 63% of the global reduction, respectively [5,6].

Measles morbidity and mortality arise in countries, such as Senegal (as evidenced by the measles epidemic episode in 2009), with sub-optimal vaccine coverage. Thus a laboratory-based surveillance system of measles was established in Senegal since 2000.

The purpose of the present study was to analyse the data collated between 2002–2012 in order to define the epidemiological profile of measles in Senegal, and secondly, to perform a molecular epidemiological analysis of of measles strains circulating in Senegal in the last 5 years.

## Methods

### Study design and setting

Senegal is situated at the extreme western part of Africa, on the Atlantic Ocean, with an estimated population of 12.9 million persons in 2011 [7]. The climate is Sudano-Sahelian characterized by an alternance of dry season (from November to May) and rainy season (from June to October).

The first measles vaccination campaign took place in 1965 [8] in response to the high mortality caused by the disease. Later in 1986, the health authorities implanted the WHO Expanded Program on Immunization (EPI) targeting several childhood diseases (including measles). Alongside this vaccination program, a virological surveillance of measles was established and since 2002 Senegal has a National Reference Center for measles located at the Institut Pasteur de Dakar. It is a case-based surveillance with laboratory confirmation for all suspected measles cases.

Once in every quarter of the year, the laboratory is required to send 10% of the total samples received to the Regional Reference Center of Ivory Coast for re-testing.

### Case definition and clinical specimen

The WHO standard measles case definition was adopted [9]. A suspected case of measles was defined as a person with fever, rash, and one of the following: cough, coryza, or conjunctivitis. The present study includes every child (from 9 months of age and above) and adult; the clinical specimens (sera) were collected between January 2004 and December 2013. Physicians used individual case investigation forms to collect data on suspected cases (age, sex, address, number of measles vaccine doses received, and the date of last measles vaccination). Serum samples were collected within 30 days of the rash onset for laboratory testing. Sera were shipped at a controlled temperature (4°C) and processed immediately on arrival at the laboratory for enzyme-linked immunosorbent assay (ELISA) test. Aliquots of samples were also stored at −20°C for molecular studies.

### Serological studies

Serological confirmation of suspect cases was made by detection of measles immunoglobulin M (IgM) antibody with an ELISA test with WHO-recommended kits (Enzygnost Anti-Measles-Virus/IgM from Dade Behring or Siemens) using manufacturers’ instructions [9]. A measles laboratory-confirmed case was defined by an anti-measles IgM absorbance value above 0.2 of the blank value. An equivocal case is defined as a sample with a difference in absorbance value (ΔA) between 0.1 and 0.2, which repeats upon retesting.

### Molecular studies

For measles virus RT-PCR (reverse transcription polymerase chain reaction) and genotyping, a 526 base pair (bp) hypervariable fragment encoding the C-terminal end of the nucleoprotein (N) was targeted in a Nested RT-PCR approach and then sequenced.

The viral RNA was first extracted directly from 200 μl of clinical specimen using the QIAamp Viral RNA kit (QIAGEN, Valencia, CA, USA) according to the manufacturer’s instructions. Each RNA sample was eluted with 60 μl nuclease-free water and stored at −80°C until amplification by RT-PCR.

The cDNA synthesis was performed using the SuperScript II Reverse Transcriptase (RT) enzyme kit (Invitrogen), the reverse primer MN5 (5'-GCCATGGGAGTAGGAGTGGAAC-3') and 5 μl of extracted RNA in accordance with the kit instructions. After synthesis, the cDNA product could be used directly for the next step (PCR amplification) or stored at −80°C until use.

For targeted fragment amplification, the first PCR was carried out in a 25 μl reaction volume containing 5 μl of template cDNA diluted at 1:5, 2.5 μl 10x reaction buffer, 1 μl of MN5 reverse primer (20 μM), 1 μl of MN6 (5'-CTGGCGGCTGTGTGGACCTG-3') forward primer (20 μM), 0.9 μl MgCl_2_ (50 mM), 0.5 of unit of Platinium Taq enzyme (Invitrogen), 0.5 μl dNTP mix (10 mM each), and 14 μl nuclease free water. The reaction mixture was amplified in a thermocycler under the following conditions: an initial denaturation step of 1 min 30 sec at 94°C followed by 35 cycles at 94°C for 30 sec, 55°C for 1 min, and 72°C for 1 min followed by a final step at 72°C for 5 min.

The Nested PCR was performed on the resulting diluted (1:50) 660 bp amplicon. The reaction mixture contained 5 μl of diluted PCR product, 5 μl 10x buffer, 2 μl of Nf1alt5 (5'-CGGGCAAGAGATGGTAAGGAGGTCAG-3') forward primer (20 μM), 2 μl of NR7alt1 (5'-AGGGTAGGCGGATGTTGTTCTGG-3') reverse primer (20 μM), 1.8 μl MgCl_2_ (50 mM), 0.5 of unit of Platinium Taq enzyme (Invitrogen), 1 μl dNTP mix (10 mM each), and 33 μl nuclease free water and the cycling conditions were 1 min 30 sec at 94°C followed by 35 cycles at 94°C for 30 sec, 58°C for 1 min, and 72°C for 1 min, and a final step at 72°C for 5 min.

PCR products were visualized by electrophoresis on 1.5% (w/v) agarose gel with molecular weight markers (100 bp ladder, New England Biolabs). Samples with 526 bp second round amplicons were bidirectionally sequenced on an ABI PRISM BigDye Terminator v3.1 Ready Reaction Cycle Sequencing kit (Applied Biosystems) on a 96-capillary ABI PRISM 3730-XL (Applied Biosystems) at Beckman Coulter Service and data in FASTA format were sent to the laboratory for analysis.

Partial nucleocapsid gene sequences were aligned against related sequences retrieved from GenBank and MeaNS (Measles Nucleotide Surveillance) database [10] using the BioEdit Sequence alignment Editor [11]. MEGA version 5 [12] was used for constructing Maximum Likelihood Tree using the Tamura-Nei evolutionary model with neighbor-joining using 100 bootstrap replicates, bootstrap replicates with values ≥70 are shown.

### Vaccination coverage data

Vaccination coverage data are kindly transmitted to us by the Direction de la Prevention, a structure based at the Ministry of Health which is in charge of the immunization in Senegal.

### Statistical analysis

We compared the distribution of laboratory-confirmed measles cases into the different age groups to verify whether the associated rates were statistically supported. The chi-squared test performed with STATA/SE software version 11.0 was used, and a *P value* < 0.05 was considered statistically significant.

### Ethical considerations

The surveillance protocol of measles was approved in its guidelines by the Senegalese National Ethical Committee of the Ministry of Health and finalized with the support of Pasteur Institute in Dakar and the WHO in the perspective of the measles elimination policy. Anonymized data were collected as part of routine surveillance. The information provided to participants was an informal description of the study. Specimens were collected, only after informed consent was granted, verbally, to local health care workers by the patients or parents in the case of minors. Throughout this study, which was specifically approved by Senegalese National Ethical Committee of the Ministry of Health, the database was shared with the Epidemiology Department at the Senegalese Ministry of Health and Prevention for appropriate public health actions. For the surveillance activities, written consent is judged not necessary by the Senegalese national ethics committee. Therefore, the consent was verbal. The patients included in this study were of all ages and were consulted by local health care workers for a cutaneous rash. Until now, written consent for surveillance activities is not judged necessary by the national ethics committee.

## Results

### Clinicodemographic characteristics of suspected measles cases

**Fig 1:**
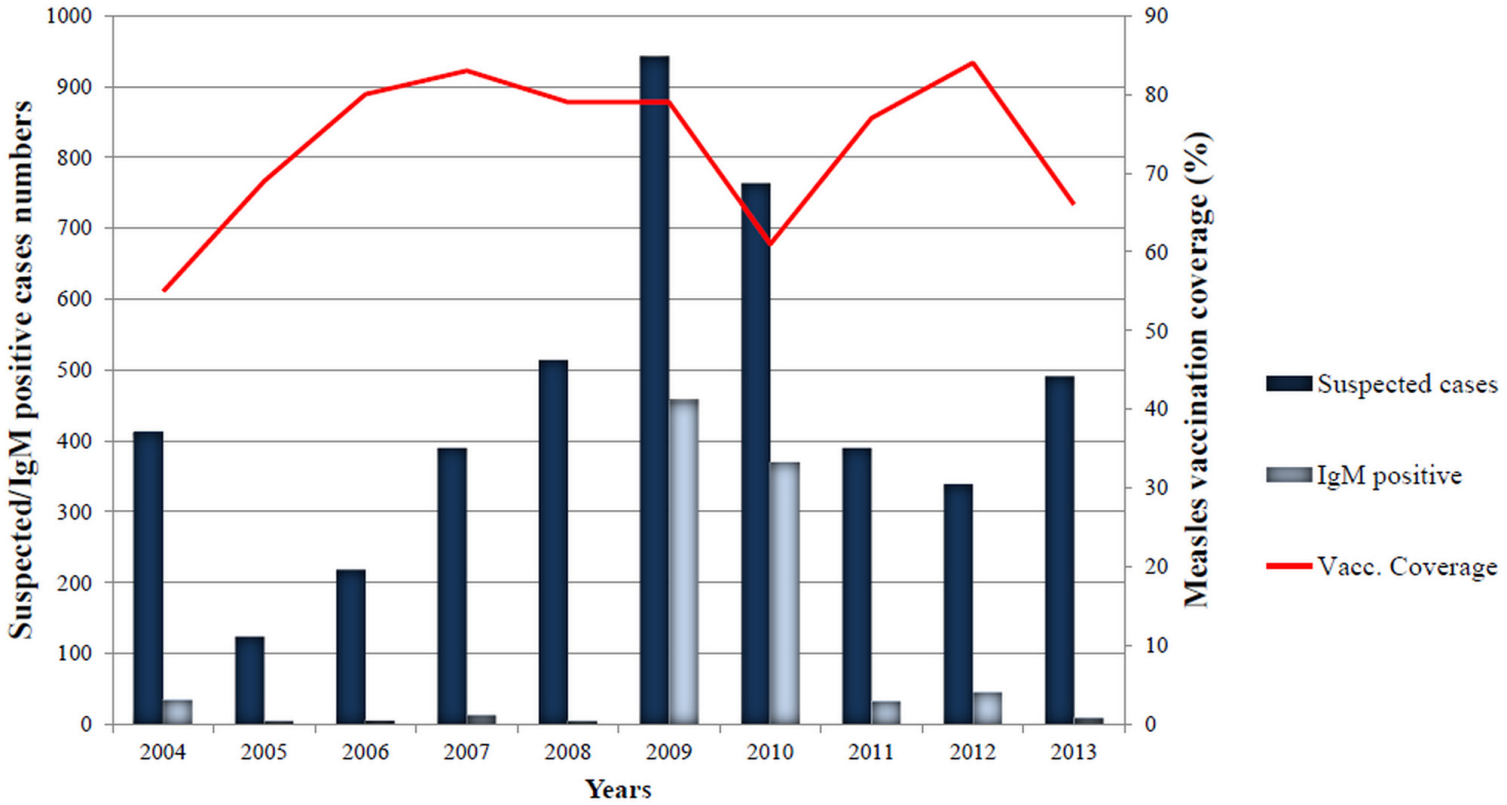
Suspected and serologically confirmed cases by and measles vaccination coverage between 2004 and 2013 in Senegal.

From 2004 through 2013 period, a total of 4580 samples were collected from suspected cases, with the most cases between 2008 and 2010 (Fig 1); 2219 cases (48.4%). Indeed in 2009, Senegal experienced a measles epidemic with continuous virus transmission from late 2009 to early 2010. During the period of study (2004–2013), collected suspected cases patient ages ranged from 9 month to 77 years (median = 6 years 2 months) and the male to female ratio was 1.2 (2108 [46%] females and 2472 [54%] males) (Table 1).

**Table 1:** Demographical characteristics, symptoms and measles serology confirmation results by age group.

		Suspected cases	Gender Male	Confirmed cases	Gender Male	Undetermined
**Characteristics**						
	**Age group**					
	0–1 years	494 (10.8%)	309 (62.5%)	148 (30.0%)	78 (52.7%)	18 (3.6%)
	2–3 years	1062 (23.2%)	578 (54.4%)	323 (30.4%)	165 (52%)	27 (2.5%)
	4–6 years	1330 (29.0%)	709 (53.3%)	231 (17.4%)	134 (58%)	32 (2.4%)
	7–15 years	1304 (28.5%)	658 (50.5%)	148 (11.3%)	81 (54.7%)	39 (3.0%)
	>15 years	292 (6.4%)	170 (58.2%)	115 (39.4%)	73 (63.5%)	15 (5.1%)
	Age Missing	98 (2.1%)	48 (49.0%)	16 (52.2%)	10 (62.5%)	2 (2.0%)
	**Total**	**4580 (100%)**	**2472 (54.0%)**	**981 (21.4%)**	**541 (55.1%)**	**133 (2.9%)**
	**Clinical signs**	**Fever**	**Conjontivitis**	**Rhinitis**	**Cutaneous rush**	
	N (%)	3952 (86.3%)	2043 (44.6%)	2864 (62.5%)	4580 (100%)	
	Missing (%)	400 (8.7%)	445 (9.7%)	405 (8.8%)	0 (0%)	

All enrolled patients presented with cutaneous rash, the primary inclusion criterion. Although they were not always reported, fever is noted in 3952/4580 [86.3%] of the patients, rhinitis in 2864/4580 [62.5%] and conjunctivitis in 2043/4580 [44.6%].

The majority of suspected cases were in children from 4–6 years (29%), 7–15 years (28.5%) and 2–3 years (23.2%). 10.8% are ≤1 year and 6.4% are ≥15 years.

### Serology analysis

Of the 4580 samples tested, 981 (21.4%) were measles laboratory-confirmed by IgM ELISA (Table 1). From these confirmed cases, 457 (46.6%) were from 2009, and 370 (37.7%) were from 2010 (Fig 1). The serology measles confirmation per year was very high during 2009–2010 periods (48.5% for each year). For the other years, the laboratory measles confirmation rates varied from 13.6% for 2012 to 0.9% in 2008. A total of 133 (2.9%) equivocal cases (named undetermined in the Table 1) were encountered during this surveillance period.

Laboratory-confirmed cases ages range from 1 to 71 years with 6 years and 4 month as mean age. The male to female ratio was 1.2 (440 [44.8%] females and 541 [55.2%] males) (P = 0.177). Regarding age groups, the highest measles IgM-positivity rate occurred among persons aged over 15 years with 19.4% (115/292) followed by 2–3 years old age group with 30.4% (323/1062), 30% (148/494) in children under one year old group, 17.4% (231/1330) in 4–6 years old group and 11.3% (148/1304) in 7–15 years old group (Table 1). The differences in proportions of cases by age were statistically significant when analysis was restricted to measles laboratory-confirmed cases only (P<0.000).

**Fig 2:**
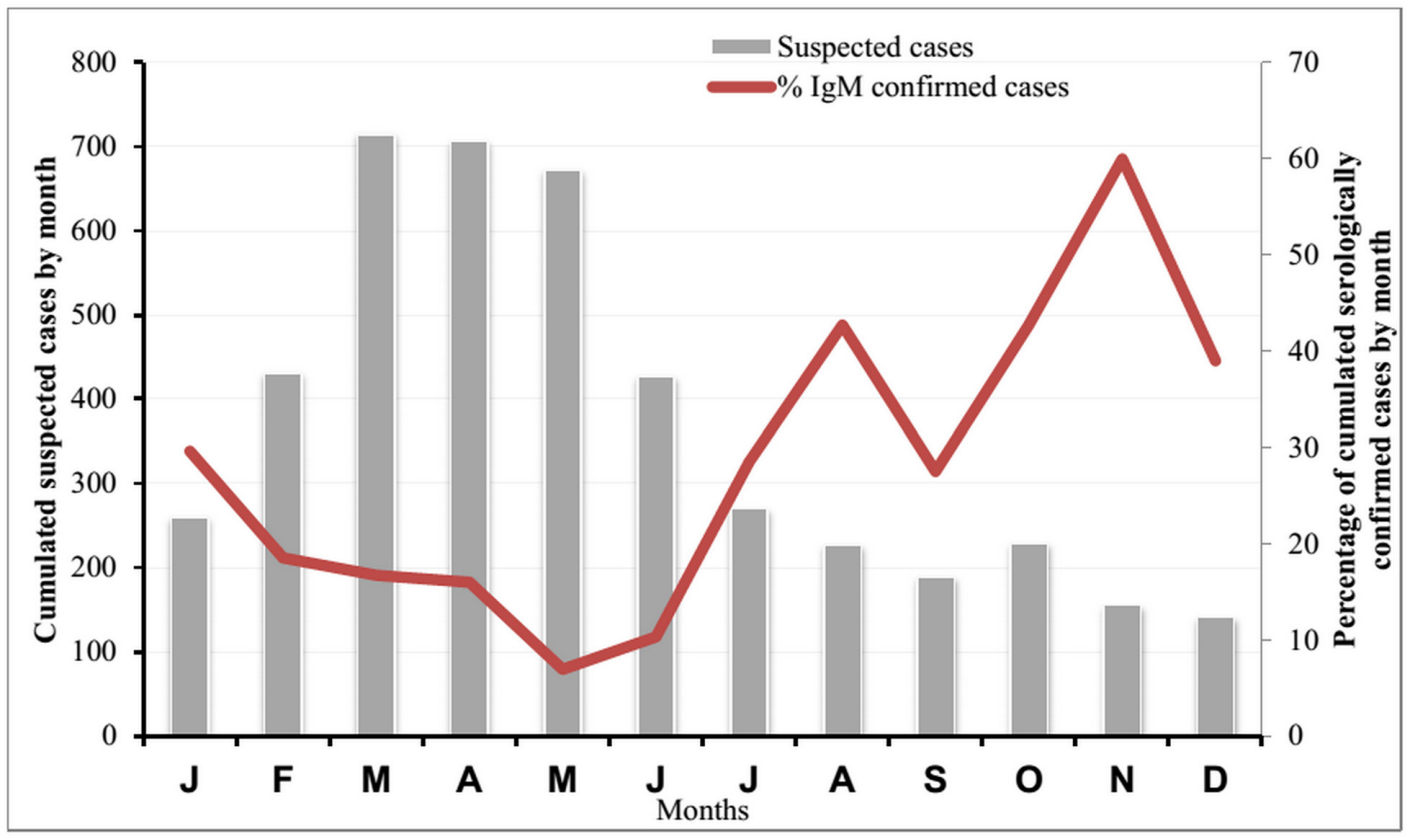
Suspected and serologically confirmed measles cases cumulated by month between 2004 and 2013 in Senegal.

In order to evaluate the circulation pattern of measles virus in Senegal (seasonality), Fig 2 presents cumulative data comprising suspected cases number for each month during the study period (2004–2013), and their confirmed cases. The majority of suspected cases were collected between February and June with a peak on March (715 cases) and April (706 cases). In the confirmed cases, the lowest rates occurred during these months (between 7% in May and 19% in February). Paradoxically, confirmed cases rates increased from July (29%) and reached a peak on November with 60% of positive patients.

### Molecular characterization of measles strains between 2009 and 2012 in Senegal

Between 2009 and 2012, sequences of the 526 nucleotides in the 3’ hypervariable region of the N gene were obtained from 29 sera samples: 11 from 2009, 10 from 2010, 5 from 2011 and 3 from 2012 samples. With the exception of 3 samples (SEN-DAK-10-013, SEN-DAK-10-053, SEN-DAK-10-361), typeable samples are within 1–3 days of rash onset. We failed to amplify the partial N gene fragment from the previous surveillance years due to poor quality of the clinical specimens. The sequences reported in this study have been deposited into the Measles Nucleotide Surveillance database (MeaNS;[10]) following WHO nomenclature recommendations (Table 2), a tool used to track measles sequence diversity and monitor elimination of virus strains: http://www.who-measles.org/Public/Web_Front/main.php.

**Table 2:** MeaNS nomenclature of genotyped measles virus from Senegal and demographical characteristics of originated patients.

Date of onset	Sample collection Date	Patient age	District location	Laboratory number	MeaNS Name	
18/07/2009	20/07/2009	8 years	DAKAR	SEN-DAK-09-360	MVs/DAKAR.SEN/29.09/16	
22/07/2009	22/07/2009	12 years	DAKAR	SEN-DAK-09-368	MVs/DAKAR.SEN/30.09/16	
03/08/2009	04/08/2009	3 years	DAKAR	SEN-DAK-09-422	MVs/DAKAR.SEN/32.09/	
22/08/2009	23/07/2009	unknown	DAKAR	SEN-DAK-09-373	MVs/DAKAR.SEN/30.09/27	
22/08/2009	23/07/2009	4 years	DAKAR	SEN-DAK-09-376	**-**	
25/08/2009	27/07/2009	8 months	DAKAR	SEN-DAK-09-390	-	
26/08/2009	27/07/2009	2 years	DAKAR	SEN-DAK-09-374	-	
27/08/2009	27/07/2009	4 years	DAKAR	SEN-DAK-09-389	MVs/DAKAR.SEN/31.09/27	
27/08/2009	27/07/2009	9 months	DAKAR	SEN-DAK-09-392	-	
22/08/2009	23/07/2009	5 years	DAKAR	SEN-DAK-09-379	-	
24/08/2009	27/07/2009	5 years	PIKINE	SEN-PIK-09-386	MVs/PIKINE.SEN/30.09/27	
21/12/2009	30/12/2009	1 year	DAKAR	SEN-DAK-10-013	MVs/DAKAR.SEN/52.09/5	
10/01/2010	19/01/2010	4 years	DAKAR	SEN-DAK-10-053	MVs/DAKAR.SEN/2.10/26	
19/02/2010	22/02/2010	7 years	DAKAR	SEN-DAK-10-235	MVs/DAKAR.SEN/8.10/53	
28/02/2010	01/03/2010	2 years	DAKAR	SEN-DAK-10-259	MVs/DAKAR.SEN/9.10/21	
14/04/2010	16/04/2010	9 months	DAKAR	SEN-DAK-10-473	MVs/DAKAR.SEN/16.10/24	
25/05/2010	25/05/2010	14 months	DAKAR	SEN-DAK-10-622	MVs/DAKAR.SEN/22.10/	
17/06/2010	18/06/2010	20 years	DAKAR	SEN-DAK-10-640	MVs/DAKAR.SEN/25.10/5	
30/10/2010	04/11/2010	13 months	DAKAR	SEN-DAK-10-783	MVs/DAKAR.SEN/44.10/2	
01/09/2010	03/09/2010	4 years	DAKAR	SEN-DAK-10-759	MVs/DAKAR.SEN/36.10/	
15/03/2010	22/03/2010	7 years	DAKAR	SEN-DAK-10-361	MVs/DAKAR.SEN/12.10/39	
31/01/2010	31/01/2011	3 years	DAKAR	SEN-DAK-11-025	MVs/DAKAR.SEN/6.11/10	
01/03/2010	04/03/2011	23 years	DAKAR	SEN-DAK-11-051	MVs/DAKAR.SEN/10.11/11	
27/02/2011	28/02/2011	5 years	DAKAR	SEN-DAK-11-059	MVs/DAKAR.SEN/9.11/6	
19/03/2011	21/03/2011	4 years	DAKAR	SEN-DAK-11-125	MVs/DAKAR.SEN/12.11/12	
08/06/2011	10/06/2011	19 years	DAKAR	SEN-DAK-11-307	MVs/DAKAR.SEN/24.11/6	
06/04/2012	06/04/2012	18 months	DAKAR	SEN-DAK-12-112	MVs/DAKAR.SEN/14.12/6	
06/05/2012	07/05/2012	9 months	DAKAR	SEN-DAK-12-225	MVs/DAKAR.SEN/18.12/8	
11/05/2012	12/05/2012	1 year	DAKAR	SEN-DAK-12-227	MVs/DAKAR.SEN/19.12/8	

**Fig 3:**
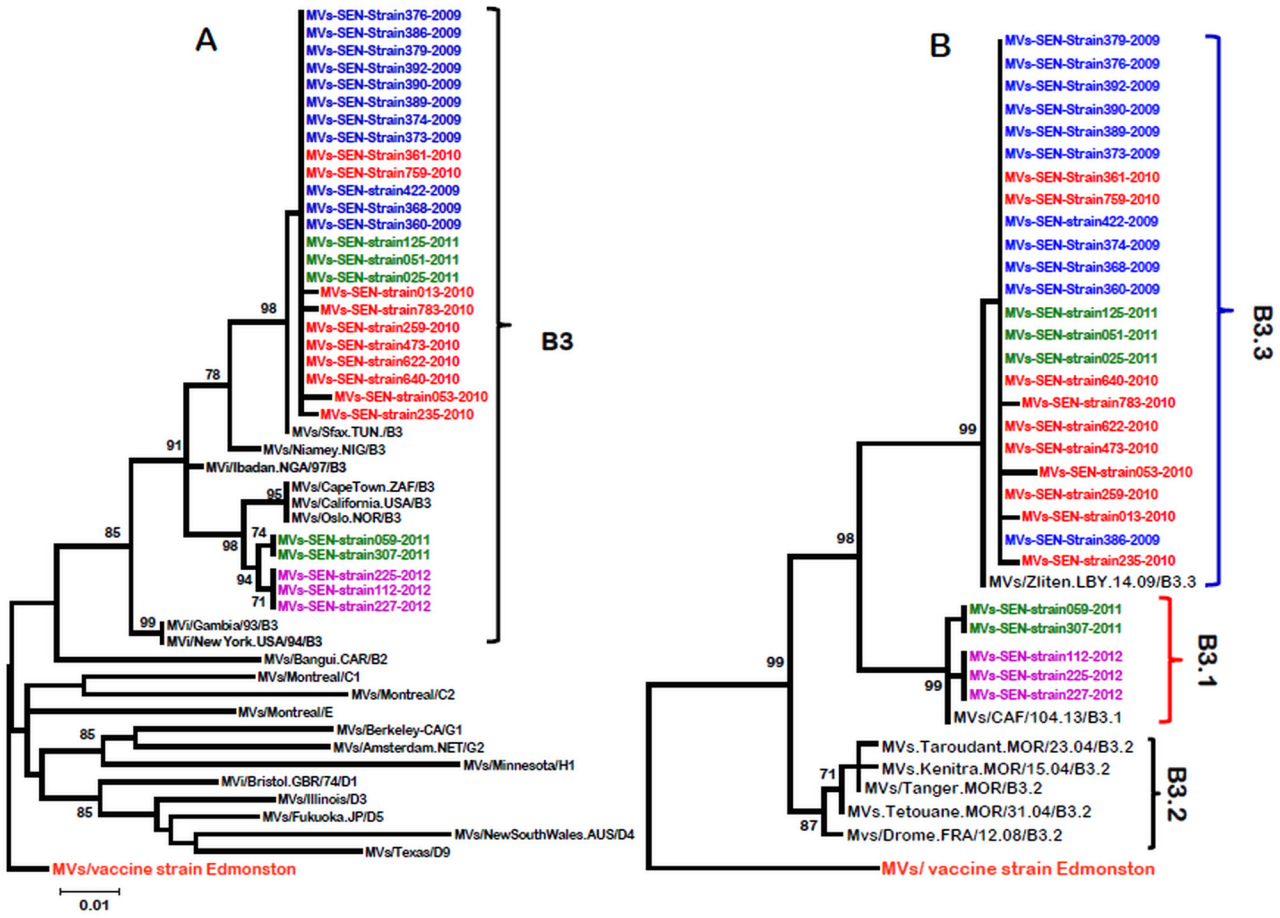
Phylogenetic analysis of strains from Senegal. Phylogenetic analyses based on the 526 nucleotides of the N gene C-terminus variable fragment obtained from 29 measles confirmed cases from Senegal between 2009 and 2012. Trees were prepared using Mega 5 version software and the neighbor-joining method with 1000 bootstrap replicates, and only bootstrap values over 70 are shown. Sequences from Senegal MeV strains are compared with the WHO reference sequences that are recommended for genotype identification (**3A**) and with the sequences of different B3 genotype subtypes retrieved on GenBank (**3B**). Senegalese MeV strains are highlighted in different colors.

For the phylogenetic analysis, measles strains from different genotypes are included in order to evaluate the genetic variability of strains originating from Senegal (Fig 3). Phylogenetic trees showed that all sequences from Senegalese strains belong to the B3 genotype cluster as confirmed by the presence of NY.USA 94 and Ibadan.NIE 97, the reference sequences for genotype B3 (Fig 3A). As genotype B3 has been divided into 3 clusters [13], sequences from the 29 Senegalese MeVs were compared to the sequences of other B3 sub-genotypes (B3.1, B3.2 and B3.3 sub-genotypes) retrieved from GenBank. The phylogenetic distribution shows that strains from Senegal are clustered in B3.1 and B3.3 sub-genotypes according to a temporal parameter. Indeed, the results show that all strains from 2009 and 2010 (21 strains) belong to the B3.3 subgroup (Fig 3B). The 3 strains from 2012 are all from the B3.1 subgroup, while those of 2011 are divided into the 2 sub-genotypes (3 in the B3.3 group and 2 in the B3.1 one).

## Discussion

The present paper described the epidemiology of measles virus in Senegal since 2004 after the implementation of a laboratory case confirmation-based surveillance system. During 2004–2012 measles surveillance, a total of 4580 suspected cases were collected and laboratory tested in Senegal, with an overall measles IgM-positivity rate of 21.4 and 2.9% as equivocal results. This positivity rate is low compared with the 40% obtained with the measles surveillance data in 40 African countries from 2002 and 2009 [14]. Although, if we consider only the laboratory-confirmed cases as it is in the results used in our analyses (and not epidemiologically linked and clinically compatible cases), this rate is slightly similar to the Senegalese serology data: around 18%. The highest rates were reported in others studies in the world, for example, Wairagkar and collaborators [15] reported 62.8% of IgM-positive samples in a retrospective study between 2005 and 2010 in India. However, it is clear that the rates are much lower in the WHO Europe region despite some outbreaks, such as those in Bulgaria in 2009 [16], in Romania in 2010 [17], in Germany [18], more recently in Ireland [19] and England in 2102 [20] or in the Netherland in 2013 [21], among other large outbreaks. Indeed, efficiency of measles surveillance system coupled with high vaccination coverage (despite an immunity gap in the population in some areas) has resulted in a dramatic decrease in measles incidence in the European Region [22]. Reduced rates were also reported in other regions such as in Taiwan with 10.5% of measles cases confirmation between 2010 and 2011 [23].

The median age of confirmed measles cases was approximately 48 months, which is 1 year greater than that of the African continent in pre-vaccine and post-vaccine eras [14]. The age distribution of MV-confirmed globally shows that more than 72% of patients were less or 6 years of age (60% between 1 to 3 years old). This result is consistent with the 78% of confirmed cases found in 9 months to 9 years (60% between 9 months to 4 years old) in Africa by Goodson et al. [14], 2011 and Wairagkar et al [15] who found similar rates in India. In industrialized countries, rates are largely lower. For example, in 2013 Europe confirmed cases rate from 0 to 4 years old is 32%, with the largest proportion of cases in Romania [24]. These findings support that measles infection remains high among children less than 5 years of age in African countries despite the efforts for an increase in vaccination coverage during the last years. Globally, African countries didn’t achieve the goal of 90% of measles vaccine coverage as recommended in the WHO and UNICEF Global Immunization Vision and Strategy for 2006–2015 [25]. For Senegal, the highest national measles vaccine coverage was reached in 2012 with 84% of the coverage (Fig 1). High vaccination coverage (≥90%) with two doses of measles vaccines is crucial for measles eradication in Africa; therefore, every opportunity should be used to reach children with routine vaccination, especially in rural areas with difficult access.

With over a third (39.4%) of the patients with measles, >15 years aged group shows the highest measles confirmation rate. Also, adults have emerged as a susceptible group for measles. The weak performance of routine EPI over the years and unvaccinated older age groups should explain this situation. This gap immunization was reported elsewhere [22].

The discrepancy observed between the number of suspected cases and confirmed cases per month during this period of the study shows that other etiologies (viral or bacterial) are probably involved in the classical cutaneous rash in Senegal. Thus, it would be important to investigate further viral etiologies such as varicella, herpetic viruses, enteroviruses, parvovirus B19 and even arboviruses for a better understanding of the epidemiology of eruptive fever syndrome in Senegal. The seasonality of laboratory-confirmed measles cases indicated measles transmission throughout the year in Senegal with a drastically increase in magnitude from July reaching a peak in November. However, these findings are largely influenced by the 2009 outbreak. Based on the studies done elsewhere [15, 26], measles transmission pattern shows variations between geographic areas.

The data about MV genotypes in Senegal from 2009 to 2012 indicated that genotype B3 is the only circulating with two distinct lineages (B3.1 and B3.3). The temporal circulation of these two lineages suggests that the epidemic episode of 2009 was caused by the B3.3 lineage; this strain would have circulated alone until early 2011 in Senegal, while B3.1 lineage should have emerged in the second half of 2011 and circulated during 2012. Previous studies have shown that measles viruses from clade B are endemic in sub-Saharan Africa [13] and B3 genotype was particularly characterized in several countries (such as Ghana, Gambia, Nigeria, Libya and Tunisia), elsewhere in Europe (such as France and Germany) and in the USA [27]. However, unlike in Senegal and in many other African countries, studies in Europe show a great diversity of genotypes in the same country, such as the United Kingdom, Germany and Spain [28], despite the fact that data on circulating genotypes is still scarce in Africa. For Senegal, a more exhaustive measles strains genotyping work between 2011 and 2013, and prior to the 2009 outbreak is necessary for a better understanding about the circulation history (endemicity, importation or co-circulation) of these B3 lineages.

However, our findings are subjected to several limitations. Firstly, vaccination was rarely documented by the physicians, which greatly limited us in our discussion. It would be interesting to know the vaccination status of confirmed cases in order to assess the weaknesses of the expanded immunization program in Senegal. People with uncertain vaccination status or unknown history of the disease should have their immunity status checked and be vaccinated accordingly. Hospitalization data or deaths cases are also lacking. Another limitation is that we only focused on the laboratory confirmed cases and did not include epidemiologically linked and clinically compatible confirmed cases, thus creating a bias in the accuracy of an epidemiological assessment of measles disease morbidity over the surveillance in Senegal. Measles strains genotyping should also be extended to the early years of surveillance for a better understanding of the molecular epidemiology of measles in Senegal.

## Conclusion

Despite these limitations, our study gives a global analysis of the available data on measles surveillance in Senegal. The results showed that children under 6 years of age are still the most vulnerable group though adults (more than 15 years of age) showed the highest absolute number of confirmed measles case, which suggests the possibility of novel measles transmission patterns (from adults to unimmunized children). The study also confirms the presence of the endemic sub-Saharan genotype (B3) in Senegal during 2009 and 2012 period. Considering the present study, improvements in the measles surveillance in Senegal are required and the introduction of oral fluid and FTA cards as an alternative to transportation of sera should be investigated to improve surveillance. The introduction of a national vaccine database including number of doses of measles-containing vaccine will greatly improve efforts to interrupt and ultimately eliminate measles virus transmission in Senegal.
